# IMMUNOHISTOCHEMICAL DETECTION OF L CELLS IN GASTROINTESTINAL TRACT MUCOSA OF PATIENTS AFTER SURGICAL TREATMENT FOR CONTROL OF TYPE 2 DIABETES MELLITUS

**DOI:** 10.1590/0102-672020210002e1651

**Published:** 2022-06-17

**Authors:** Priscila Costa ESTABILE, Mara Cristina de ALMEIDA, Eduardo Bauml CAMPAGNOLI, Marco Aurelio SANTO, Marcos Ricardo da Silva RODRIGUES, Fábio Quirillo MILLÉO, Roberto Ferreira ARTONI

**Affiliations:** 1 Postgraduate Program in Science in Gastroenterology, University of São Paulo, São Paulo, SP, Brazil;; 2 Department of Structural, Molecular and Genetics Biology, State University of Ponta Grossa, Ponta Grossa, PR, Brazil;; 3 Department of Dentistry, State University of Ponta Grossa, Ponta Grossa, PR, Brazil;; 4 Department of Medicine, State University of Ponta Grossa, Ponta Grossa, PR, Brazil;; 5 Institute for Applied Research in Medicine, INSPAM, Ponta Grossa, Brazil;; 6 Associate Professor at University of São Paulo School of Medicine, is Director of Bariatric and Metabolic Surgery Unit at Hospital das Clinicas, Brazil.

**Keywords:** Type 2 Diabetes Mellitus, Bariatric Surgery, Obesity, Incretins, Glucagon-Like Peptide-1, Diabetes Mellitus Tipo 2, Cirurgia Bariátrica, Obesidade, Incretinas, Peptídeo 1 Semelhante ao Glucagon

## Abstract

Type 2 diabetes mellitus (T2DM) is a disease of global impact that has led to an increase in comorbidities and mortality in several countries. Immunoexpression of the incretin hormones such as glucagon-like peptide-1 (GLP-1) and peptide YY (3-36) (PYY_3-36_) can be used as a scorer in the gastrointestinal tract to analyze L-cell activity in response to T2DM treatment.

**OBJECTIVE:**

This study aimed to investigate the presence, location, and secretion of L cells in the small intestine of patients undergoing the form of bariatric surgery denominated adaptive gastroenteromentectomy with partial bipartition.

**METHODS:**

Immunohistochemical assays, quantitative real-time polymerase chain reaction (qPCR), and Western blot analysis were performed on samples of intestinal mucosa from patients with T2DM in both the preoperative and postoperative periods.

**RESULTS:**

All results were consistent and indicated basal expression and secretion of GLP-1 and PYY_3-36_ incretins by L cells. A greater density of cells was demonstrated in the most distal portions of the small intestine. No significant difference was found between GLP-1 and PYY_3-36_ expression levels in the preoperative and postoperative periods because of prolonged fasting during which the samples were collected.

**CONCLUSION:**

The greater number of L cells in activity implies better peptide signaling, response, and functioning of the neuroendocrine system.

## RESUMO - RACIONAL

O diabetes tipo 2 (DM2) é uma doença de impacto mundial que tem levado ao aumento de comorbidades e mortalidade em vários países. A imunoexpressão dos hormônios incretínicos glp-1 e pyy^3-36^, pode ser usada como marcador no trato gastrointestinal para analisar a atividade da célula L em resposta ao tratamento do DM2.

**OBJETIVO:**

O presente estudo teve como objetivo investigar a presença, localização e secreção de células L no intestino delgado de pacientes submetidos à forma de cirurgia bariátrica denominada gastroenteromentectomia adaptativa com bipartição parcial.

**MÉTODOS:**

Ensaios imunohistoquímicos, reação quantitativa em cadeia de polimerase em tempo real (qPCR) e análise de manchas ocidentais foram realizados em amostras de mucosa intestinal de pacientes com diabetes tipo 2 nos períodos pré- e pós-operatório.

**RESULTADOS:**

Todos os resultados foram consistentes e indicaram expressão basal e secreção de peptídeos glucagon-1 (GLP-1) e peptídeos YY (PYY3-36) incretinas por células L. Uma maior densidade de células foi demonstrada nas porções mais distais do intestino delgado. Não foi encontrada diferença significativa entre os níveis de expressão GLP-1 e PYY3-36 nos períodos pré-operatório e pós-operatório, provavelmente devido ao estado de jejum prolongado durante o qual as amostras foram coletadas

**CONCLUSÃO:**

O maior número de células L em atividade implica melhor sinalização de peptídeo, resposta e funcionamento do sistema neuroendócrino.

## INTRODUCTION

Type 2 diabetes mellitus (T2DM) is a multifactor metabolic disorder usually associated with obesity and abdominal fat, dyslipidemia, arterial hypertension, and cardiovascular disease ^9^. Obesity is a severe health problem worldwide, affecting over 650 million adults and 124 million children and adolescents ^27^. One of the main causes of this panorama is the sudden change in modern lifestyle, such as sedentary lifestyle, consumption of highly energetic and low fiber foods (i.e., foods rich in carbohydrates and lipids in the daily consumption of the diet), and their association, which only worsens the case of obesity and T2DM ^10^.

T2DM treatment recommendations include dietary and lifestyle changes, oral hypoglycemic drugs, exogenous insulin, and more recently digestive tract surgery intervention ^19^ ^,^ ^25^. The lack of success of clinical treatment in combating obesity and diabetes has led to the development of surgical procedures for the treatment of metabolic syndrome and T2DM ^5^. Patients with obesity have a suppressed incretin effect and a consequent imbalance of glycemic homeostasis. Bariatric surgery has the potential of T2DM control in up to 90% of patients with severe obesity due to caloric restriction, improvement of insulin resistance, pancreatic beta-cell function, and the incretin effect of glycogen-like protein^1^. Santoro^24^ found that glycemia can be effectively controlled in patients with T2DM following gastroenteromentectomy. Surgical techniques demonstrate different responses, but all of them contribute to better glycemic control when compared to clinical treatment ^26^. In a general review with 621 articles, T2DM remission is confirmed, which can reach 80% in patients undergoing bariatric surgery ^4^.

In this current scenario, metabolic surgery has taken a considerable role in weight loss, contributing to metabolic control, and showing an improvement in the state of obesity and related comorbidities. We already know that conservative treatment has been failed in 80% of obese patients, while 80% of obese patients who have undergone metabolic surgery are successful in long-term weight loss and resumption of metabolic functionality, showing better results than drug therapy or only lifestyle change ^18^.

This adaptive procedure, which is aimed at neuroendocrine improvements instead of gastrointestinal restriction and malabsorption ^20^, leads to increased serum levels of the incretins (hormones that stimulate a decrease in blood glucose levels), such as glucagon-like peptide-1 (GLP-1) and peptide YY (3-36) (PYY_3-36_), in postprandial patients 5 years following surgery ^24^. These findings underscore the importance of incretins in the control of T2DM. According the study by Nauck and Meier, observed clinical improvement in obese patients after bariatric surgery results in the consequence of the early passage of food in the gastrointestinal tract and in turn stimulates the mucosa by increasing the production of enteral hormones, such as GLP-1 and GIP, which contribute to the improvement of glycemic control and satiety at the hypothalamus level, and these effects are reflected in improved diabetic status and obesity ^22^. These hormones play key roles in stimulating the secretion of insulin by the endocrine pancreas ^15^. It is therefore of considerable importance to understand the secretion mechanisms of epithelial intestinal cells as well as the identity and location of cells responsible for the action of these peptides ^3^. In the intestinal epithelium, we found cells that release incretins in the response to the nutrients in contact with intestinal lumen called L cells ^6^. L cell found in the distal ileum and large intestine secretes GLP-1 and PYY_3-36_ which promote delayed gastric emptying and act as a satiety signal to improve glycemic control^12^.

This study aimed to investigate the presence, location, and secretion of L cells in the small intestine of patients undergoing adaptive gastroenteromentectomy with partial bipartition.

## METHODS

The present study received approval from the Human Research Ethics Committee of the State University of Ponta Grossa (Brazil) under process no. 0783/10 (register 37/2010).

Seven patients submitted to AGPB (Adaptative Gastroenteromentectomy with Partial Bipartition - Partial Duodenal Switch) study, with a body mass index >35 kg/m^2^, T2DM with difficult clinical control, and dietary and medical treatment for a minimum of 2 years, were included in the study. The screening of patients and surgical treatment were carried out at the *Hospital Vicentino da Sociedade Beneficente São Camilo* in the city of Ponta Grossa (state of Paraná, Brazil).

### Acquisition of samples

Mucosa from the stomach and duodenum was collected through digestive endoscopy, and mucosa from the ileum was collected through colonoscopy, which was performed 10-15 days before surgery as well as 3 months after surgery. During surgery, samples of mucosa were collected from the small intestine on the site to perform the gastro-ileal anastomosis 260 cm from the ileocecal valve.

The samples submitted to quantitative real-time polymerase chain reaction (qPCR) and Western blot analysis were stored in RNA Later solution (Qiagen) and maintained at a temperature of −80°C. Samples to be submitted to immunohistochemical analysis were placed in Bouin solution (75 mL of picric acid, 20 mL of formaldehyde, and 5 mL of acetic acid) for 24 h and stored in 70% ethanol at 4°C until use.

### Immunohistochemical analysis

The tissue to be analyzed was embedded in paraffin. Serial histological sections measuring 5 μm in thickness were cut on manual rotary microtome (Leica RM2125RT). The sections were dehydrated in an alcohol series, stained with hematoxylin and eosin, and mounted on slides.

To determine the immunohistochemical reactions, the histological sections were fixed on silanized slides (3-aminopropyl-triethoxysilane, Sigma) and placed in an oven at 56^o^C for 24 h. The sections were then cleared twice in xylene at room temperature for 10 min per process and hydrated in decreasing concentrations of ethanol (100%, 90%, 70%, and 50%), followed by a distilled water bath. Specific antigen recovery was performed for each antibody used. Table 1 lists the antibodies and respective technical details.

**Table 1 -:** Antibodies used with respective clone, brand, concentration, and antigen retrieval method.

Antibody	Clone	Brand	Dilution	Antigen retrieval
GLP-1	SC 57166 (monoclonal)	Santa Cruz Biotechnology	1:1000	Sodium citrate buffer; microwave
PYY_3-36_	SC 98995 (polyclonal)	Santa Cruz Biotechnology	1:1000	Sodium citrate buffer; microwave

GLP-1: glucagon-like peptide-1; PYY_3-36_: peptide YY (3-36).

Antigen retrieval was performed in a microwave oven at full power for 20 min. A citrate buffer solution (10 mM citric acid, pH 6.0) was used for antigen recovery. The samples were left at room temperature to cool for 20 min. The histological cuts were then washed in running water for 5 min and incubated in 20 volumes of aqueous hydrogen peroxide solution changed once in every 5 min (totally six times) to block endogenous peroxidase. A further 5-min washing in running water was performed, and the sections were then washed three times (2 min per wash) with phosphate-buffered saline (PBS). The histological sections were incubated with the primary antibodies (previously diluted in PBS) for approximately 18 h (overnight) at 4°C. The dilution of the primary antibodies was 1:1000.

The sections were then washed three times with PBS (3 min per wash), and the reaction was revealed using the Novo Link Polymer Detection System (Novocastra, UK). Incubation was performed with Post Primary Block for 30 min. The sections were washed in TBS for 2 × 5 min, followed by incubation with NovoLink Polymer for 30 min and washing in TBS for 2 × 5 min, with gentle rocking. Peroxidase activity was developed with a DAB working solution for 5 min. The slides were rinsed with water, and the sections were counterstained with Carazzi hematoxylin, cleared in xylene, and mounted with Canada balsam. For the negative control, slides containing histological sections underwent all steps of the immunohistochemical reaction except incubation with the primary antibodies.

The histological sections were analyzed using bright field microscopy (Olympus BX41) with a digital image capture system (Olympus DP71 equipped with DP-Controller software program). The images were treated using the Image Pro Plus 6.0 program. To estimate the frequency of immunoreactive cells, photomicrographs of the slides were taken, and the number of cells was counted in 10 random unit areas (1 area = 1000 μm^2^) of the mucosal layer in each section for all patients.

### qPCR

Total RNA was extracted from approximately 3 mm^3^ of target tissue (mucosa of the distal ileum) from each patient using the Illustra RNAspin Mini RNA Isolation Kit (GE Healthcare^®^), following the manufacturer’s instructions. Approximately 1 μg of total RNA was used for cDNA synthesis (First-Strand cDNA Synthesis Kit - GE Healthcare^®^). The cDNA samples were tested for the genetic expression of GLP-1 and PYY_3-36_ by qPCR in a Stratagene Thermal Cycler Mx3005P, using the Brilliant II SYBR^®^ Green QPCR Master Mix (Stratagene^®^) with commercial primers (QuatinTect Primer Assay - Qiagen).

The amplifications were performed for GLP-1 and PYY_3-36_ genes in duplicate PCRs as follows: 1 μL of cDNA (15 ng); 1 μL of commercial primers (1x) - mix forward and reverse; 12.5 μL of Sybr Green (1x); 0.375 μL of ROX dye (30 nM); and 10.125 μL of water, resulting in a total volume of 25 μL for each sample to be tested. The samples were preheated to 95^o^C for 10 min, followed by 40 cycles of 95^o^C for 15 s, 60^o^C for 1 min, and 72^o^C for 15 s and ending with one cycle for the disassociation curve: 95^o^C for 1 min, 55^o^C for 30 s, and 95°C for 30 s.

The level of expression of each target gene was normalized to the level of expression of the ribosomal 18S gene. The cycle threshold (Ct) was measured, and a relative change in the expression level of one specific gene was presented as 2^−^ΔΔ^Ct^ (Livak and Schmittgen, 2001).

### Western blotting

Frozen samples of target tissue were ground in a lysis solution [Tris-HCl (20 mM), NaF (10 mM), NP40 (1%), NaCl (150 mM), and SDS (0.1%)]. Total proteins of the cell homogenate were assayed using the BCA Protein Assay Reagent kit (Thermo Scientific) with ELISA, followed by reading in a UV mini-1240 spectrophotometer. The samples were boiled in buffer solution (glycerol, mercaptoethanol, 10% SDS, 10N NaOH, and bromophenol) for 5 min to prevent the formation of disulfide bonds in the protein.

Based on the molecular weight, the protein samples were separated in 10% polyacrylamide gel. The protein bands were transferred to a nitrocellulose membrane (Hybond; Amersham Pharmacia Biotech, London, UK). The blocking reaction was performed with a 2% bovine serum albumin in TBS-T buffer (20 mM TRIS, pH 7.5, 0.5 M NaCl, 0.1% Tween 20) to prevent nonspecific binding. The membrane was subjected to detection by overnight incubation with the primary antibody (Table 1). Following the reaction, incubation of the secondary antibody and protein detection were performed. The revelation was carried out using the ECL-Plus kit (Amersham Pharmacia Biotech, Piscataway, NJ, USA), with the material exposed in a dark room and revealed with X-rays.

### Statistical analysis

The tissue samples were compared among the preoperative, transoperative, and postoperative periods and with different sections of the gastrointestinal tract. The results were evaluated by the paired Student’s t-test with the significance level set to 5% (p<0.05) using the GraphPad Prism 5.0 program ^21^.

## RESULTS

**Figure 1 -:**
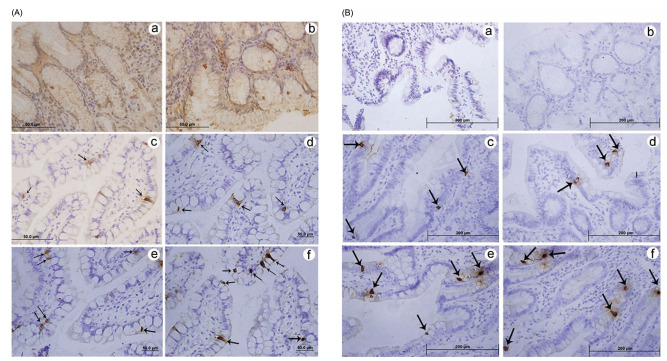
Immunolabeling using the (A) polyclonal antibody for peptide YY (3-36)and (B) monoclonal antibody for glucagon-like peptide-1. In both panels, note the absence of labeled immune L cells and a background in the region of the gastric fundus (a) and pylorus (b); few immune cells labeled in the region of the jejunum (c) and proximal ileum (d); and greater frequency of labeled active immune L cells in the most distal portions of the ileum (e; f). The paired Student’s t-test was used with the significance level set to 5% (p<0.05).

The analysis of the intestinal tract from the jejunum to the distal ileum revealed a growing number of active L cells (Figure 1A and 1B, Table 2).

**Figure -2:**
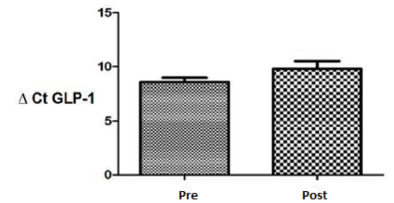
mRNA expression levels of glucagon-like peptide-1 from the ileum tissue in preoperative (pre) and postoperative (post) periods. Bands above the columns indicate gene expression verified by western blotting. The paired Student’s t-test was used with the significance level set to 5% (p<0.05).

**Figure 3 -:**
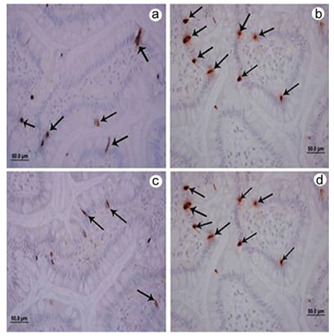
Immunolabeling using the monoclonal antibody for glucagon-like peptide-1. Note the increased number of labeled L cells in the postoperative period (b and d) compared to the preoperative period (a and c). The paired Student’s t-test was used with the significance level set to 5% (p<0.05).

**Figure 4 -:**
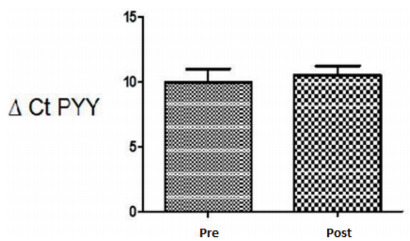
mRNA expression levels of peptide YY (3-36) from the ileum tissue in preoperative (pre) and postoperative (post) periods. The paired Student’s t-test was used with the significance level set to 5% (p<0.05).

**Figure 5 -:**
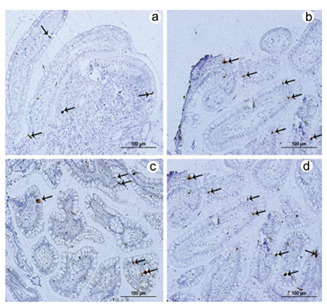
Immunolabeling using the polyclonal antibody for peptide YY (3-36). Note the increased number of labeled L cells in the postoperative period (b and d) compared to the preoperative period (a and c). The paired Student’s t-test was used with the significance level set to 5% (p<0.05).

L cells had the same secretion and basal expression (statistically not significant) under the conditions studied, as observed in patients with 12 h of fasting and confirmed by immunohistochemical analysis, Western blotting, and qPCR (Figures 2 and 3) for GLP-1. The expression of the PYY_3-36_ gene was confirmed by immunohistochemical analysis and qPCR (Figures 4 and 5).

**Table 2 -:** Total number of L cells per field marked by glucagon-like peptide-1 (GLP-1) and peptide YY (3-36) (PYY_3-36_) antibodies in portions of gastrointestinal tract (GIT) during surgery.

TGI	PYY_3-36_	GLP-1
Jejunum	4	3
Proximal ileum	3	4
Medial ileum	5	7
Distal ileum	7	7

Comparison of number of L cells among portions of TGI for GLP-1 and PYY_3-36_ antibodies.

However, no significant differences were found between the fasting preoperative and postoperative periods of AGPB for GLP-1 (p=0.1669) and PYY_3-36_ (p=0.0017) gene expression, as determined by qPCR. The immunohistochemical data demonstrated a greater number of L cells stained with GLP-1 and PYY_3-36_ antibodies in the postoperative period (p=0.0043).

## DISCUSSION

The present study offers the first mapping of intestinal L cells in patients undergoing surgical AGPB treatment for T2DM. In such cases, immunohistochemical analysis was employed to detect the expression of the incretins GLP-1 and PYY_3-36_.

The present data lend support to the hypothesis that although L cells are dispersed throughout the gastrointestinal tract, a greater concentration is found in the distal portion of the small intestine ^3^.

Immunohistochemistry has been employed to locate GLP-1 in the gastrointestinal tract of mice, pigs, and humans ^7^. The same is true for PYY_3-36_ ^17^. However, previous studies have identified the location of L cells employed immunohistochemistry to detect the incretin GLP-1, along the human gastrointestinal tract from the stomach to the distal ileum. According to these studies, when investigating the gastrointestinal tract of cadavers by immunohistochemistry with incretins (GLP-1) depending on the region of the intestine, there will be a difference in L-cell density according to the distal jejunum and ileal portion, in comparison with the duodenum and proximal jejunum, and an increasing density of the colon proximal to the distal with higher levels in the rectum, due to the cell presenting a basal level of expression ^13^.

In addition, Jorsal ^13^ observed that the presence of immunoreactive L-cell activity will also vary if the individual is healthy or has T2DM because his stimulus areas occurred in more proximal segments in the case of healthy individuals, or more distal segment in case of T2DM patients, demonstrating the plasticity suffered by the activity of the enteroendocrine L cell according to the local sensitization at an epithelial site.

Enteroendocrine L cell is found along the gastrointestinal tract (GIT), and its distribution varies according to the small intestine segment. The hormone GLP-1, an incretinic secretion released by it, acts on glycemic homeostasis and satiety control, that is, investigating the density and location of the L cells is of great relevance for better understanding of the metabolic profile and T2DM control. ^13^

GLP-1^2^ and PYY_3-36_ ^1^ have a short half-life, and their expression fluctuates throughout the day in accordance with food intake, while the physiological secretion of GLP1 and PYY_3-36_ occurs in response to food stimuli ^8^.

A number of bariatric procedures have been successful in the control of satiety and the improved metabolic control, ^14^ ^,^ ^16^ ^,^ ^23^which demonstrates the importance of the procedure regarding the control of T2DM. According to the study by Milléo et al.^19^, the reduction of the gastrointestinal tract contributed to an improved postprandial neuroendocrine response, allowing more nutrients to be absorbed in the distal portion of the small intestine and increasing the secretion of GLP-1 and PYY_3-36_, measured by the ELISA method.

The events responsible for weight loss or management are closely linked to food intake and gastrointestinal hormone expression response, which will influence the metabolic balance of individuals. However, it is of great importance that after bariatric surgery procedure, maintenance of enterhormone rates such as GLP-1 and PYY_3-36_ causes physiological changes in the individual; hence, reflex of the plasticity incretinous expression of the L cells in the body can be observed, showing transmutation and metabolic adaptation for a homeostatic balance of the same ^20^.

## CONCLUSION

The findings of the present study suggest the importance of the role of GLP-1 and PYY_3-36_ in the standardization and regulation of T2DM, as evidenced by the labeling of active intestinal L cells in the most distal portions of the gastrointestinal tract. An increased number of active L cells result in better peptide signaling, response, and function of the neuroendocrine system.
